# Protective Effects of Polyphenol Enriched Complex Plants Extract on Metabolic Dysfunctions Associated with Obesity and Related Nonalcoholic Fatty Liver Diseases in High Fat Diet-Induced C57BL/6 Mice

**DOI:** 10.3390/molecules26020302

**Published:** 2021-01-08

**Authors:** Ahtesham Hussain, Jin Sook Cho, Jong-Seok Kim, Young Ik Lee

**Affiliations:** 1 Lee’s Biotech Co., Ltd. #416, C Dong, 17 Techno 4-ro, Yuseon-gu, Daejeon 34013, Korea; ahtsham07@gmail.com (A.H.); nabucco1@hanmail.net (J.S.C.); 2 Myunggok Medical Research Institute, College of Medicine, Konyang University, Daejeon 35365, Korea; jskim7488@konyang.ac.kr

**Keywords:** plants extract, *Rubus crataegifolius*, *Crataegus pinnatifida* Bunge, *Cinnamomum cassia*, obesity, metabolic dysfunctions, NAFLD, HFD-induced mouse model

## Abstract

Background: Currently, obesity is a global health challenge due to its increasing prevalence and associated health risk. It is associated with various metabolic diseases, including diabetes, hypertension, cardiovascular disease, stroke, certain forms of cancer, and non-alcoholic liver diseases (NAFLD). Objective: The aim of this study to evaluate the effects of polyphenol enriched herbal complex (*Rubus crataegifolius*/ellagic acid, *Crataegus pinnatifida* Bunge/vitexin, chlorogenic acid, *Cinnamomum cassiaa*/cinnamic acid) on obesity and obesity induced NAFLD in the high-fat diet (HFD)-induced obese mouse model. Methods: Obesity was induced in male C57BL/6 mice using HFD. After 8 weeks, the mice were treated with HFD+ plants extract for 8 weeks. Body weight, food intake weekly, and blood sugar level were measured. After sacrifice, changes in the treated group’s liver weight, fat weight, serum biochemical parameters, hormone levels, and enzyme levels were measured. For histological analysis, tissues were stained with hematoxylin-eosin (H&E) and Oil Red-O. Results: Our results showed that the herbal complex ameliorated body weight and liver weight gain, and decreased total body fat in HFD-fed animals. Post prandial blood glucose (PBG) and fasting blood glucose (FBG) were lower in the herbal complex-treated group than in the HFD control group. Additionally, herbal formulation treatment significantly increased HDL levels in serum and decreased TC, TG, AST, ALT, deposition of fat droplets in the liver, and intima media thickness (IMT) in the aorta. Herbal complex increased serum adiponectin and decreased serum leptin. Herbal complex also increased carnitine palmityl transferase (CPT) activity and significantly decreased enzyme activity of beta-hydroxy beta methyl glutamyl-CoA (HMG-CoA) reductase, and fatty acid synthase (FAS). Conclusions: The results of this study demonstrated that the herbal complex is an effective herbal formulation in the attenuation of obesity and obesity-induced metabolic dysfunction including NAFLD in HFD-induced mouse model.

## 1. Introduction

Obesity results from an imbalance between energy intake and expenditure due to excessive consumption of foods rich in fat and carbohydrates [1]. It is associated with various diseases, including cardiovascular diseases, hypertension, diabetes, non-alcoholic liver diseases (NAFLD), and certain forms of cancer. All of these diseases are associated with hyperglycemia, insulin resistance, high cholesterol, and fatty liver [1,2]. Common drugs like simvastatin is used for the treatment and prevention of obesity by reducing cholesterol. Statins, a common group of cholesterol-lowering pharmaceuticals, effectively decrease low-density lipoprotein (LDL) cholesterol by inhibiting beta-hydroxy beta methyl glutamyl-CoA (HMG-CoA) reductase. However, they have been found to have adverse side-effects, and severe complications. Rhabdomyolysis, one of the severe adverse effects from statin on muscle and its statin-associated side effects have also reported [3,4]. Therefore, considering the high prevalence of adverse side effects associated with the drugs used for its management, it is important to find a safe alternative formulation for the control of obesity and associated metabolic dysfunctions including NAFLD.

The development of herbal products for the treatment of obesity is of global interest. There is increasing number of natural products, including *Rubus crataegifolius*, a type of red raspberry native to Korea, Japan, and China. *R. crataegifolius* is widely used as a traditional medicine for treating impotence, spermatorrhea, enuresis, asthma, allergy, and inflammation [5,6,7]. It contains various bioactive compounds such as phenolic acids, triterpenoids, flavonoids, and ellagitannin, and antioxidants such as anthocyanins, epicatechin, ellagic acid, and ferulic acid. It has been also reported to contain anti-rheumatism, anti-oxidant, and anticancer agents [8,9].

Hawthorn (*Crataegus pinnatifida* Bunge), a prevalent fruit tree in China, belongs to the Rosaceae family. *Crataegus pinnatifida* has been used as a traditional medicine in Asia (Korea, China, and Japan) [10], in treating hyperlipidemia and chronic heart failure, and it is also used in various digestive ailments [11,12]. Its main bioactive components are chlorogenic acid, epicatechin, vitexin, rutin, and hyporoside, which are involved in the treatment of various diseases, including hypertension, cardiovascular, anti-oxidative, atherosclerosis, and hyperlipidemia [11,12,13,14]. Also, previous studies reported that hawthorn has anti-obesity effects that help in reducing plasma levels of triglycerides and LDL [12].

Cinnamon (*Cinnamomum cassia*) is one of the most important spices obtained from the inner bark of several tree species from the genus Cinnamomum. In Arabian and European countries, it is used in sweet and savory foods. The aqueous extract of Cinnamon is known for insulin-regulation and glucose utilization via enhancing the insulin signaling pathway [15,16] and preventing high-fructose diet-induced insulin resistance [17]. Cinnamon is used as a traditional medicine, and it is known for its hepatoprotective [18], anti-oxidant [19], anti-obesity [20], anti-hyperlipidemia [21], and anti-diabetic [17,22,23] activities.

In the present study, we evaluated the anti-obesity and related metabolic dysfunctions including NAFLD of plants extract, using a high-fat diet (HFD)-fed obese mouse model. We used plants extract alone or combination of two (Rubus, Crataegus:RC) or three herbs (Rubus, Crataegus, and Cinnamon: RCC’). The combined plants extract showed good result in lowering obesity in the mouse. Besides, we investigated whether HFD + RC and HFD + RCC’ exert a promoting effect on metabolic dysfunctions including NAFLD. We also investigated the mode of action of plants extract, their hepatoprotective effect especially in lipid metabolism, and anti-atherogenic effects in HFD-fed mice.

## 2. Results

### 2.1. 2-D HPLC Chromatogram of Three Plants Extract

**Figure 1:**
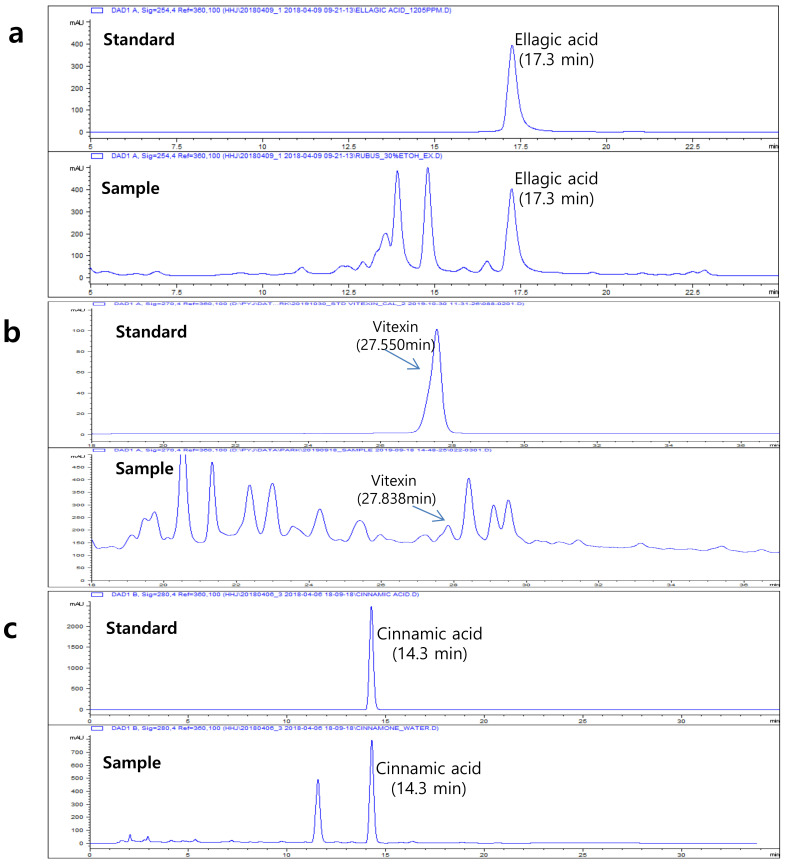
2-D HPLC chromatogram of three plants extract: (**a**) Ethanol extract of *Rubus coreanus* (ellagic acid), (**b**) ethanol extract of *Crataegus pinnatifida* Bunge (vitexin), and (**c**) water extract of *Cinnamomum cassia* (cinnamic acid).

The Rubus, Crataegi, and Cinnamon extracts were standardized by determining the amounts of ellagic acids, vitexin, and cinnamic acids, using HPLC chromatography. *Rubus crataegifolius* extracts were monitored at 280 nm for ellagic acid; 0–35 min, 10–12%; 35–38 min, 12–100% (Figure 1a). Crataegus extracts were monitored at 270 nm for Vitexin; 0–30 min, 20–50% B; 30–40 min, 50–100% (Figure 1b). Cinnamon extracts were monitored at 270 nm for Cinnamon acid; 0–20 min, 0–10%; 20–35 min, 12–15%; 35–40 min, and 15–100% (Figure 1c). From these experimental results ellagic acid, vitexin and cinnamic acid were used for the standard index of these 3 plants.

### 2.2. Treatment with Plants Extract Decreased Total Body Weight, Food Intake, Fat Tissue, and Liver Weight in HFD-Induced Obese Mice

To evaluate whether each plants (R,C) and complex plants (R + C, R + C + C’) extracts has an anti-obesity effect, we measured the body weight weekly, throughout the 8 weeks treatment period. At the end of the 8th week, the body weight of HFD-fed mice was significantly (*p* < 0.05) higher as compared to that of animals in the normal group (ND), indicating effective HFD-induced obesity in the mice. The body weight, food intake, liver weight, and perirenal fat, abdominal fat, epididymal fat and total fat weight were significantly higher (*p* < 0.05) in HFD-fed mice compared to those in the normal diet (ND) group (Table 1). However, treatment of HFD-fed animals with HFD + R, HFD + C, HFD + RC, and HFD + RCC’ significantly (*p* < 0.005) attenuated body weight gain, total fat tissue and liver weight (Table 1). Whereas, treatment with HFD + SMV significantly (*p* < 0.005) decreased total body weight gain as compared to the HFD-fed animals. Collectively, our results showed that plant formulation (R,C,R + C, R + C + C’) can effectively prevent body weight gain and fat deposition in HFD-fed mice.

### 2.3. Effects of Plant Extracts on Biochemical Parameters

**Figure 2:**
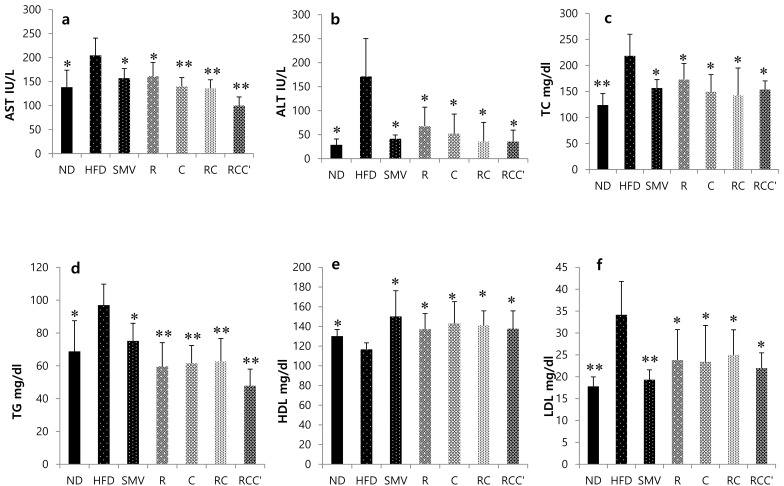
Effect of plants extract on biochemical parameter in HFD-fed C57BL/6 mice (**a**) aspartate transaminase (AST); and (**b**) alanine transaminase (ALT). (**c**) Total cholesterol (TC); (**d**) triglyceride (TG); (**e**) high-density lipoprotein (HDL); (**f**) low-density lipoprotein. Data were represented as mean ± SD (*n* = 7). The results were compared to the HFD group using the Student’s *t*-test (** corresponds to *p* < 0.005, * corresponds to *p* < 0.05). The error bars represent the SD.

After 8 weeks of treatment, in comparison to those in ND, HFD-fed mice had significantly (*p* < 0.05) elevated levels of serum AST (aspartate aminotransferase), ALT (alanine aminotransferase), TC (total cholesterol), TG (triglyceride), and LDL (low-density lipoprotein) (Figure 2a,b,c,e), and a significantly (*p* < 0.05) lower level of serum HDL (high-density lipoprotein) (Figure 2e). However, treatment of HFD-fed mice with HFD + R, HFD + C, HFD + RC, or HFD + RCC significantly (*p* < 0.05) attenuated the levels of serum AST, ALT, TC, TG, and LDL, and similar results were seen in treatment with HFD + SMV. Additionally, the HDL levels were significantly (*p* < 0.05) higher in HFD + R, HFD + C, HFD + RC, or HFD + RCC (Figure 2e) treated animals. Similarly, the HFD + SMV treated mice showed a significantly (*p* < 0.05) higher level of serum HDL.

### 2.4. Effects of Plant Extracts on Blood Sugar in HFD-Induced Obese Mice

**Figure 3:**
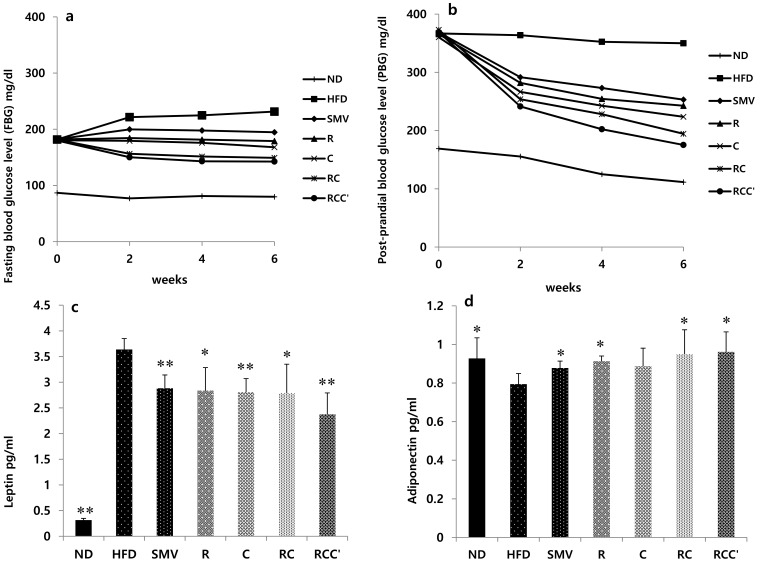
Effect of plant extracts on blood sugar at 2, 4, and 6 weeks and hormone level in C57BL/6J mice. (**a**) Fasting blood sugar (FBG) at 2, 4, and 6 weeks, (**b**) Post-prandia blood glucose (PBG) at 2, 4, and 6 weeks, (**c**) leptin and (**d**) Adiponectin. Statistical difference was determined using the independent samples *t*-test. Value are represented as mean ± SD (*n* = 7). The results were compared to the HFD group using the Student’s *t*-test (** corresponds to *p* < 0.005, * corresponds to *p* < 0.05). The error bars represent the SD.

FBG was tested after 8 h of starvation and PBG was tested 2 h after 1.0 g/kg glucose was orally administered. Compared to the ND group, the HFD group had elevated levels. However, the FBG level was attenuated in HFD-fed animals treated with HFD + R, HFD + C, HFD + RC, HFD + RCC, or SMV (Figure 3a). PBG level was attenuated in HFD-fed animals treated with HFD + R, HFD + C, HFD + RC, or HFD + RCC’, and a similar result was observed in HFD-fed mice treated with SMV (Figure 3b). The result indicated that herbal formulations affect FBG and PBG.

### 2.5. Effects of Plant Extracts on Hormone in HFD-Induced Obese Mice

To evaluate the changes in hormone found in the serum of HFD + plant formulation or HFD + SMV treated animals, the leptin and adiponectin hormones were analysis by ELISA (Figure 3c,d). The leptin level significantly (*p* < 0.05) decreased in HFD + R, HFD + C, HFD + RC, and HFD + RCC treated groups as compared with the HFD-fed control group. However, a similar result was seen in treatment with HFD + SMV (Figure 3c). In contrast, the adiponectin level significantly (*p* < 0.05) increased in HFD + R, HFD + C, HFD + RC, and HFD + RCC treated groups as compared with the HFD-fed control group. With regard to the leptin hormone, a similar profile was observed in the positive control (HFD + SMV) group compared with the HFD-fed control group (Figure 3d).

### 2.6. Effects of Plant Extracts on FAS, HMG-CoA Reductase, and CPT in HFD-Induced Obese Mice

**Figure 4:**
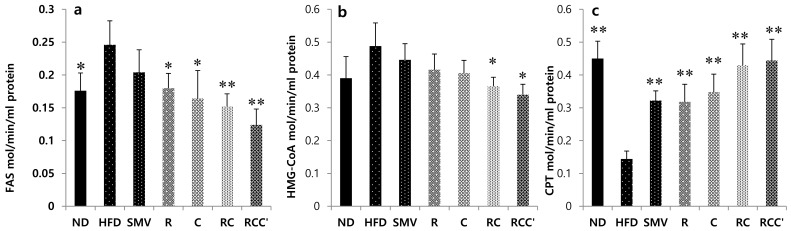
Activity of hepatic lipid regulation enzymes fatty acid synthase (FAS), beta-hydroxy beta methyl glutamyl-CoA (HMG-CoA) reductase and carnitine palmityl transferase (CPT) in C57BL/6J mice treated with plants extract. Values are represented as mean ± SD (*n* = 7). The results were compared to the HFD group using the Student’s *t*-test (** corresponds to *p* < 0.005, * corresponds to *p* < 0.05). The error bars represent the SD. (**a**) FAS (Fatty acid synthase); (**b**) HMG-CoAR (3-hydroxy-3-methyl-glutaryl-coenzyme A reductase); (**c**) CPT (carnitine palmitoyltransferase).

After 8 weeks of treatment, the experimental results showed a significant (*p* < 0.05) decrease in FAS in normal diet animals as compared with HFD-Fed animals. Even more, FAS significantly (*p* < 0.05) decreased in the HFD + R, HFD + C, HFD + RC, or HFD + RCC treated animals as compared with the HFD-fed animals. Whereas, FAS decrease in the HFD + SMV treated animals was not significant (Figure 4a). However, HMG-CoA activities significantly (*p* < 0.05) attenuated in the HFD + RC or HFD + RCC treated animals, but in HFD + R, HFD + C, or HFD + SMV treated animals, the decrease was not significant as compared with HFD-fed animals (Figure 4b). In contrast, the CPT activities significantly increased (*p* < 0.05) in the HFD + R, HFD + C, HFD + RC and HFD + RCC treated animals as compared with the HFD-fed animals. A similar result was seen in the HFD + SMV treated animals as compared with the HFD-fed control (Figure 4c). HMG-CoA and CPT activities were measured as described [24,25].

### 2.7. Plants Extract Ameliorated HFD-Induced Histological Alterations and Fat Deposition in Liver

**Figure 5:**
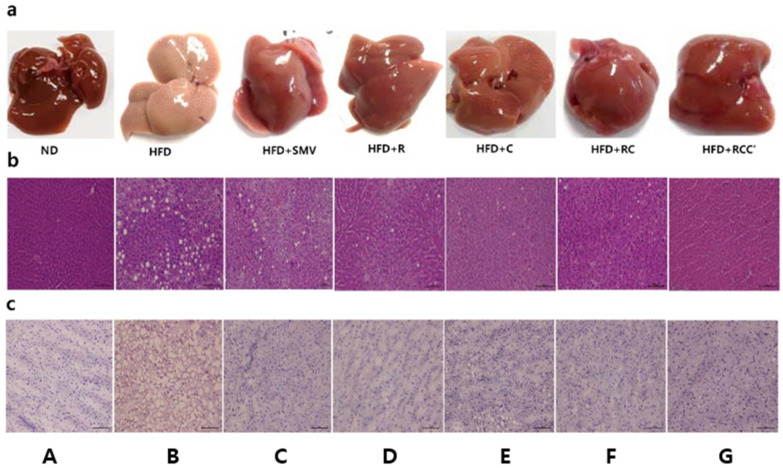
Effect of plants extract on HFD-induced histopathological changes of liver tissue stained with H&E and oil red O staining (**a**–**c**) Representative image of liver tissue of normal control animal (ND) stained with Oil red O. Representative images of the liver tissues of animals treated with (**A**) Normal diet (**B**) HFD, (**C**) HFD + SMV, (**D**) HFD + R, (**E**) HFD + C, (**F**) HFD + RC and (**G**) HFD + RCC’. Pathophysiological examination of the tissue sections was performed under a light microscopy at 200× magnification.

After treatment, liver in the ND group showed dark red color, sharp edges were determined as healthy. However, liver in the HFD-fed group with light color and irregular surface suggested fatty liver. Treatment with plants extract (HFD + R, HFD + C, HFD + RC, or HFD + RCC’) could relieve hepatic morphological damage (Figure 5a). In contrast, the histopathological evaluation of the liver tissue accompanied with hematoxylin and eosin staining paraffin tissue sections demonstrated a normal lobular architecture with the appearance of only a few small-sized fat droplets in normal-fed animals (Figure 5b). In contrast, large-sized vacuoles were observed in the liver of HFD-fed animals by a substantial deposition of fat, indicating fatty liver. The liver tissue appeared to be normal with less or no deposition of fat droplets in HFD-fed animals treated with HFD + R, HFD + C, HFD + RC, or HFD + RCC’. A similar profile was seen in animals treated with SMV. Further, histological evaluation of oil red O staining in frozen liver tissue sections of ND mice revealed the normal lobular architecture of the liver, with negligible appearance of large vacuoles (Figure 5c). Whereas, the liver of HFD-fed mice exhibited an aberrant histological structure characterized by abundant large vacuoles, indicating increased fatty liver cells and finally hepatosteatosis. However, minimal small vacuoles were observed in HFD-fed mice when treated with HFD + R, HFD + C, HFD + RC, HFD + RCC’, or HFD + SMV.

### 2.8. Plants Extract Ameliorated HFD-Induced Histological Alterations in Aorta

**Figure 6:**
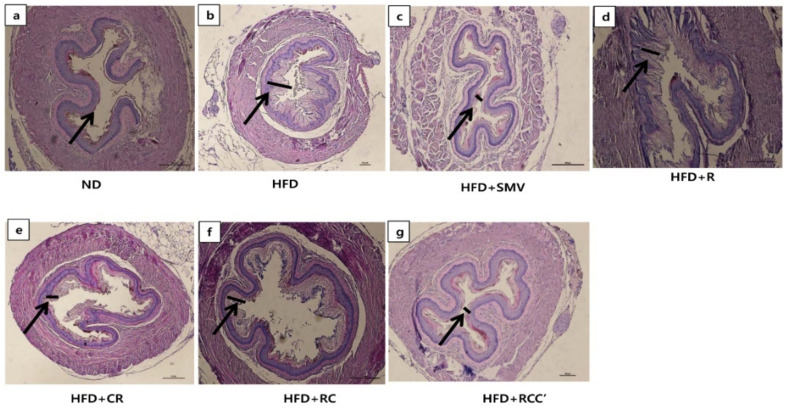
Effect of plants extract on HFD-induced histopathological changes in H&E-stained aorta tissue. (**a**) Representative image of the liver tissue of normal control animal (ND). Animals fed with (**b**) HFD, (**c**) HFD + SMV, (**d**) HFD + R, (**e**) HFD + C, (**f**) HFD + RC, and (**g**) HFD + RCC’. Pathophysiological examination of the tissue sections was performed under a light microscopy at 200× magnification.

The histopathological evaluation of the aorta tissue accompanied with hematoxylin and eosin staining paraffin tissue sections showed normal intima-media thickness (IMT) of the thoracic aortic wall of normal-fed animals (Figure 6a). In contrast, the intima thickness of the thoracic aortic wall of the HFD-fed mice showed a substantial deposition of fat (Figure 6b). However, minimal small intima thickness of the thoracic aortic wall was observed in HFD-fed mice when exposed to plants extract (HFD + C, HFD + RC, and HFD + RCC’), while, HFD + R treated animals showed little or no effect on IMT (Figure 6c–g). While in HFD + SMV treated animals, IMT of the thoracic aortic wall were similar or small amounts decrease as compared with HFD-fed animals.

### 2.9. Effect of Rubus Crataegifolius Extracts on Plasma Anticoagulation Parameters

The effect of Rubus on blood coagulation parameters (aPTT: Activated partial thromboplastin time; TT: Thrombin time; PT: Prothrombin time) are shown in a table. aPTT, TT, and PT were extended very significantly in in vitro assay (Table 2). The significant effect RF extracts had on aPTT, TT, and PT, indicates the beneficial effect of RF on intrinsic and extrinsic coagulation pathway in vitro and in vivo (Table 2).

## 3. Discussion

The excess amount of caloric intake and insufficient energy expenditure disturbs lipid metabolism and induces obesity and related metabolic dysfunctions including NAFLD [26]. According to the World Health Organization, in 2016, more that 1.9 billion adults were overweight (BMI: Body mass index > 25–29.9 kg/m^2^) and of these, over 650 million were obese (BMI > 30 kg/m^2^). Since the worldwide prevalence of obesity, the global epidemic of NAFLD is also spreading. The risk of NAFLD/NASH was from 4.1- to 14-fold higher in patients with a higher BMI. In line with these data the higher BMI is an independent, dose-dependent risk factor for fatty liver disease. The anti-obesity effect of natural products is under exploration, and this may be an excellent alternative strategy for the development of future effective and safe anti-obesity drugs [27]. Several studies demonstrated the anti-obesity and metabolic dysfunctions effect of herbal medicine formulations including DSHT [26], *Flos Lonicera* [28], *Rhizoma Atractylodis Macrocephalae* [29], *Bofu-tsusho-san* [30], and *Rehmannia glutinosa* [31]. In this study, we presented experimental results showing the anti-obesity and metabolic dysfunctions including NAFLD properties of plants extract, using HFD-induced obese mice as a model.

Animals on a HFD showed an increase in total body weight gain which ultimately resulted in obesity [32]. In this study, mice on a HFD showed significantly increased body weight than those on a normal diet, indicating the successfully execution of diet-induced obesity. However, the oral supplementation of plants extract (HFD + R, HFD + C, HFD + RC, and HFD + RCC’) administered to HFD-fed mice significantly decreased body weight, indicating the anti-obesity effect of plants extract, and combined plants extract (HFD + RC and HFD + RCC’) showed a better anti-obesity effect. A similar effect was also observed when HFD + SMV was administrated, which in line with recent reports revealing a potent inhibitory effect of SMV on body weight gain in HFD-induced obese rats and mice [33].

Obesity and NAFLD are characterized by an increase in the mass of adipose tissue and number and size of fat cells. According to the metabolic requirements of the organism, adipose tissue plays an important role in energy balance and change mass [34,35]. The main characteristic of NAFLD is excessive accumulation of fat in hepatocytes. NAFLD can range from relatively benign non-alcoholic fatty liver (NAFL) to the aggressive form called non-alcoholic steatohepatitis (NASH)), characterized by both fatty liver and liver inflammation. Since NAFL and NASH are chronic diseases, without proper treatment, they may lead to life-threatening complications such as fibrosis, cirrhosis, liver cancer or liver failure. Nonalcoholic fatty liver, the first stage of NAFLD, is defined as the accumulation of excessive fat in the liver in the absence of excessive alcohol consumption and the lack of any secondary cause. It is diagnosed in patients with visible lipid accumulation in at least 5% of hepatocytes; however, diagnosis is hampered by lack of characteristic symptom. In our experimental results showed that significant reduction in liver weight and total fat weight (intestine, kidney, and testis fat) in HFD + R, HFD + C, especially combined plants extract (HFD+ RC, and HFD+ RCC’) treated animals. These results suggested that the combined plants extract effectively attenuated proliferation or differentiation of adipose tissues.

The total fat mass directly correlated with the body weight and body weight gain; thus, it can be concluded that plants extract (HFD + R, HFD + C, and especially HFD + RC and HFD + RCC’) showed a better effect in suppressing body weight gain by repressing the expansion of adipose tissue mass.

Increased serum level of biochemical parameters AST, ALT, TC, TG, and LDL, and a pronounced decrease in serum HDL level were observed in the HFD group compared to the ND group. However, treatment with plants extract (HFD + R, HFD + C, HFD + RC, and HFD + RCC’), or HFD + SMV, showed significant reductions in AST, ALT, TC, TG, and LDL and an increase in serum HDL level, compared to HFD-Fed animals. Especially, combination of plants extract (HFD + RC, and HFD + RCC’) showed good restoration as compared to the HFD-fed animals. Based on these results, it is possible for plants extract and SMV to improve the homeostasis of lipid parameters in a HFD-fed state. Recent studies have indicated that SMV treatment results in marked improvements in serum and hepatic lipid profiles including TG, TC, AST, ALT, and HDL cholesterol in HFD-induced obese rats or mice [33,34]. There is also a strong link between obesity and T2DM. According to data collected between 1999 and 2006, the prevalence of overweight and obesity among American adults with diabetes was 80.3% and 49.1%, respectively. Our experimental results showed that mice on a HFD showed marked increase in glucose levels (PBG and FBG). However, treatment with plants extract (HFD + R, HFD + C, HFD + RC, and HFD + RCC’), showed reduced serum glucose levels in HFD-fed animals, producing a hypoglycemic effect. Thus, our study suggests the beneficial impact of plants extract, especially combination of plants extract (HFD + RC and HFD + RCC’), on glucose homeostasis and metabolism in HFD-induced obesity. Additionally, the marked improvement in the histopathological characteristics of hepatic tissue in HFD-fed mice by plants extract (HFD + R, HFD + C, HFD + RC, and HFD + RCC’), and SMV treatment further supports the anti-obesity impact of plants extract. Overall, our results revealed that treatment of HFD-fed animals with a combination of plants extract (HFD + RC, and HFD + RCC’) was more effective than treatment with either alone, at restoring liver morphology and reducing the serum levels of AST and ALT. Obesity and metabolic dysfunction such as insulin resistance or dyslipidemia are the best-known mechanisms leading to excessive accumulation of triglycerides in hepatocytes. It has been shown that obese patients are characterized by enhanced lipolysis or triglycerides and fatty acid release from adipose tissue. This excessive breakdown of triglycerides causes accumulation of fatty acids in the form of diacylglycerol not only in the liver but also in other tissues. In the case of liver, hepatic uptake of circulating fatty acids is mediated by fatty acid transporters; FATP (fatty acid transporter proteins). Once in the cytosol, fatty acid are stored in the form of triacylglycerols to be exported from hepatocytes or metabolized via oxidation. Importantly, all of these processes are disturbed in NAFLD patients leading to excessive TAG accumulation in hepatocytes.

The enzyme activities, HMG-CoA reductase, FAS, and CPT, which are involved in hepatic lipogenesis and lipolytic reactions, were measured in liver tissue [34,35,36]. HMG-CoA reductase is important in cholesterol synthase [35,36,37]. In the present study, there was an increase in the activity of FAS and HMG-CoA in the liver of HFD-fed animal, which further increased the synthesis of TG and TC and accumulation of lipid in the liver. FAS and HMG-CoA significantly (*p* < 0.05) decreased in animals treated with HFD + R, HFD + C, HFD + RC, or HFD + RCC, as compared with HFD-fed animals. The CPT activities significantly (*p* < 0.05) increased in animals treated with HFD + R, HFD + C, HFD + RC, or HFD + RCC, as compared with HFD-fed animals. A similar result was seen in HFD + SMV treated animals as compared with HFD-fed control (Figure 5c). These results demonstrated that plants extract have the potential to prevent obesity and NAFLD.

Animals on a HFD showed a significantly increased lipid profile as compared to those on a normal diet. This aggravating lipid profile indicated the presence of dyslipidemia. Dyslipidemia is a health risk factor of atherosclerosis [38,39]. In this study, LDL accumulated in the intima-media thickness (IMT) of the blood vessels in HFD-fed animals compared with the normal-fed animals. In contrast, minimal or small intima thickness of the thoracic aortic wall was found in HFD-fed mice treated with plants extract (HFD + C, HFD + RC, and HFD + RCC’), though HFD + R showed little or no effect on IMT.

Many traditionally Chinese medicines (TCMs) have been shown to have anticoagulant activity and activate blood circulation to remove blood stasis including, Danggui (*Radix angelicae Sinensis*), Honghua (*Flos carthami*), and Danshen (*Salvia miltiorrhiza Bunge*) [40]. In this study we used plants extract (*Rubus)* to determined blood clotting time in vitro. The in vitro antithrombosis effect of *Rubus crataegifolius* extracts was investigated by measuring activated partial thromboplastin time (APTT), prothrombin time (PP), and thrombin time (TT). The result indicated that *Rubus* exhibited strong anticoagulation activity. Since, *Crataegus* lowered the IMT and rubus showed strong anticoagulation activity, RC could be a good source for preventive atherosclerosis and finally cardiovascular disease (CVD) prevention. Collectively, our results showed that treatment of HFD-fed animals with a combination of plants extract (HFD + RC, and HFD + RCC’) or single plants extract (HFD + R, and HFD + C) was effective in Obesity, and NAFLD including blood circulation.

In conclusion, the results of this study demonstrated that plants extract is effective in anti-obesity and related metabolic dysfunctions including NAFLD, and blood circulation against HFD-induced obesity. Plants extract attenuate TC, TG, AST, and ALT, increase HDL levels in serum, decrease deposition of fat droplets in liver, and decrease IMT in aorta. Plants extract treatment increased the serum adiponectin and decreased serum leptin, compared with HFD-fed mice. Plant extract treatment significantly reduced the enzymatic activities of HMG-CoA reductase and FAS, while increased CPT activity. *Rubus crataegifolius* extracts themselves have the potential to exhibit anticoagulation activity. The results of this study demonstrated that plants extract is an effective herbal formulation for metabolic dysfunction including obesity and NAFLD in HFD-fed mouse.

## 4. Materials and Methods

### 4.1. Preparation of Herbal Formula

The unripened fruit of *Rubus*, stem bark of *Cinnamon* and ripened fruit of *Crataegus* were purchased from Kyung Dong Medicinal Herb Market (Seoul, Korea). These herbal samples were verified by taxonomist in Plant Resource Center, Korea Research Institute of Bioscience and Biotechnology (KRIBB, Daejeon, Korea) and are kept in the herbarium of KRIBB. For herbal extract preparation, chopped herbs were mixed with water (volume of water was10 times greater than volume of herbs) and incubated at 80 °C for 8 h. After incubation, the extract was filtered and freeze dried for 3 days. *Rubus* and *Crataegus* extracts were prepared separately with 50% ethanol for 8 h at 70 °C in a reflux apparatus, and *Cinnamon* extract was prepared separately with water for 8 h at 80–90 °C in a round bottom flasks. The extracts were filtered, concentrated under reduced pressure and freeze-dried for 3 days, and the dry powder was prepared. The yield (%) of individual extracts was 23.1%, 8.4%, and 12.8%, respectively. Then, the three kinds of powder were mixed for preparing materials for treatment.

### 4.2. Standardization of Rubus, Crataegus, and Cinnamon Analysis by HPLC

The qualification of unripened fruit of *Rubus*, fruit of *Crataegus*, and stem bark of *Cinnamomum* was carried out using a high-performance liquid chromatography analysis system (Agilent technologies 1260 infinity, Agilent Technologies, Santa Clara, CA, USA) equipped with an autosampler (G1329B) and a UV detector (G1316A). Chromatographic separation was carried out on an Agilent Zorbax SB-C18 column (150 × 4.6 mm i.d., 5 µm) at 40 °C for rubus and crataegus and 25 °C for Cinnamon, with a flow rate of 0.8 mL/min, and the injection volume was 10 μL. Water containing 0.1% trifluoroacetic acid (A) and acetonitrile containing 0.1% trifluoroacetic acid (B) were selected as the mobile phase in gradient elution. The three different HPLC gradients were as follows.

*Rubus* (ellagic acid); 0–35 min, 10–12% B; 35–38 min, and 12–100% B. The column was equilibrated for 10 min and the chromatogram was acquired at 280 nm. *Crataegus* (vitexin); 0–30 min, 20–50% B; 30–40 min, and 50–100% B. The column was equilibrated for 10 min and the chromatogram was acquired at 270 nm. *Cinnamon* (cinnamic acid); 0–20 min, 0–10% B; 20–35 min, 12–15% B; 35–40 min, and 15–100% B. The column was equilibrated for 10 min and the chromatogram was acquired at 270 nm.

### 4.3. Animal Experiments and Sample Collection

Six-week-old male C57BL/6 mice were purchased from Saeronbio (Gyeonggi-do, Korea) and acclimatized for a week before the experiments. They were kept at a constant temperature of 21 °C, a relative humidity of 55%, and under a 12 h light-dark cycle. They were maintained on a high-fat diet (HFD) with the exception of those on a normal diet (provided with a normal chow diet), for 8 weeks. After 8 weeks, they were divided into 7 groups; a normal chow diet group (ND, *n* = 7), high-fat diet group (HFD, *n* = 7), HFD + simvastatin (SMV, 10 mg/kg body weight orally through gavage, *n* = 7; positive control), HFD + Rubus (R: 200 mg/kg, *n* = 7), HFD + Crataegus (C: 200 mg/kg, *n* = 7), HFD + Rubus + Crataegus (RC: 200 + 50 mg/kg, *n* = 7), and HFD + Rubus (R) + Crataegus (C) + Cinnamon (C’) (RCC: 200 + 50 +50 mg/kg, *n* = 7). The normal chow diet consisted of food with 12.7% of the calories being derived from fat, while for HFD, 60% of the calories was being contributed by fat. The body weights of animals were measured weekly. Mice were maintained on HFD for 8 weeks and treatment was orally by gavage once a day for 8 weeks. Finally, after 16 h fasting, blood samples were collected from the mice retro-orbitally before the animals were sacrificed. Serum biochemical parameters and hormone levels were measured using enzymatic assay. The animal was sacrifice by using cervical dislocation. For histological analysis, liver tissues, aortas, and fat tissues were excised, washed rapidly, fixed in 10% neutral buffered formaldehyde, and stored until used. The animals used in this study were housed and cared for in accordance with the national institute of health (NIH) guidelines for the care and use of laboratory animals. The experimental protocol was approved by the institutional animal ethical committee, Korea Research Institute of Bioscience & Biotechnology (KRIBB-AEC-18176), Daejeon, Korea.

### 4.4. Serum Biochemistry Analysis

After 8 week of desired treatment, the blood samples were collected and kept for 1 h at room temperature to allow clotting, centrifuged for 15 min at 3000 rpm, and stored at –80 °C until used. The HDL-C, TG, TC, LDL-C, and aspartate AST/ALT levels were measured as per the manufacturer’s instructions and analyzed using the Beckman Coulter AU480 Chemistry Analyzer (Olympus-Beckman Coulter, Brea, CA, USA).

### 4.5. Serum ELISA Analysis

The serum leptin concentrations were measured using the enzyme-linked immunosorbent assay for the quantitative detection of mouse leptin (mouse Leptin ELISA Kit, Invitrogen by Thermo Fisher Scientific, Vienna, Austria). Serum adiponectin concentrations were also measured (mouse adiponectin ELISA kit, Abcam), as per manufacturer’s instructions.

### 4.6. Enzyme Activity Assay

After tissue collection, for hepatic enzymes, we used the Hulcher and Oleson (24) method with a little modification. The tissue homogenate was made using a buffer solution that contains 0.1 mol/L of triethanolamine, 0.02 mol/L of EDTA, and 2 mmol/L of dithiothreitol (pH 7.0). The homogenate was centrifuged at 600× *g* for 10 min to remove any cell debris. The supernatant was collected and centrifuged (10,000× *g*, followed by 12,000× *g* for 20 min at 4 °C) to remove the mitochondrial pellet. The supernatant was then ultracentrifuged twice (100,000× *g* for 60 min at 4 °C) to remove the cytosolic supernatant. The mitochondrial and microsomal pellets were then dissolved in 800 μL of homogenization buffer, and the protein content was determined using the Bradford method, using bovine serum albumin as the standard. Fatty acid synthase (FAS) activity was measured in the cytosolic fraction by monitoring the malonyl-coenzyme A-dependent oxidation of nicotinamide adenine dinucleotide phosphate (NADPH) at 340 nm using the method by Nepokroeff et al. [7], where the activity was represented by the oxidized NADPH nmol/min/mg protein. Carnitine palmitoyl transferase-1 (CPT) activity was determined in the mitochondrial fraction using the method by Markwell et al. [25] and the results were expressed as nmol/min/mg protein. The activity of 3-hydroxy-3-methylglutaryl-coenzyme A (HMG-CoA) reductase was measured in microsomes based on a modification of the method of Hulcher and Oleson [24], and the results are expressed as released CoA-SH nmol/min/mg protein.

### 4.7. Blood Sugar Measurement

After the desired treatments, fasting blood sugar (FBG) was tested after 8 h of starvation and post-prandial blood glucose level (PBG) was tested 2 h after 1.0 g/kg glucose was orally administered through gavage. Blood was collected from the tail vein, and the FBS was determined using a blood glucose meter (Accu-Chek Advantage, Roche, Charles Avenue, West Sussex, UK).

### 4.8. Histological Analysis of Liver and Aorta

After 8 weeks of treatments, the liver tissues and aorta were collected, fixed in 10% neutral buffered (formaldehyde), and sliced (5 μm thick) and trimmed transversely into serial sections using a cryo-microtome (Leica Biosystems, Richmond, VA, USA). Sections were later deparaffinized in xylene, rehydrated through an ascending ethanol series, and stained with hematoxylin-eosin (H&E). Oil Red-O staining was used to analyze fat deposition in liver tissues. The liver tissues were embedded in FSC 22 frozen section compound (Leica Biosystems, Richmond, VA, USA) and sectioned (5 μm thickness) using a cryostat (Tissue-Tek^®^ Cryo3^®^ Flex Microtome/Cryostat, Sakura, Leiden, The Netherlands). The sections were stained with Oil Red-O staining, and mounting was performed with xylene-based media. The slides were observed under an Olympus BX 61 microscope (Olympus, Tokyo, Japan) at 200× magnification and images were acquired using an Olympus DP70 digital camera (Olympus).

### 4.9. In Vitro Coagulation Assay

Measurements of APTT, PT, and TT were performed using a coagulometer (Amelung coagulometer KC-1A, Labcon, Germany) according to the manufacturer’s specification. Briefly, the plasma of animals was incubated with the RF extract or aspirin for 2 min at 37 °C. Then 100 μL of plasma was mixed with 100 μL of phosphatidylethanolamine in the process plate, and coagulation was initiated by adding 1 mM CaCl_2_, 100 μL of thromboplastin, and 100 μL of bovine thrombin into 100 μL of incubated plasma for APTT, PTT, and TT assay, respectively.

### 4.10. Statistical Analyses

The values are expressed as mean ± standard deviation (SD). Data are presented as means ± SD. Different letters above bars indicate significant difference at *p* < 0.05. Null hypotheses of no difference were rejected if *p*-values were less than 0.05.

## Figures and Tables

**Table 1:** The five major phenolic compounds in ADL ethanolic extract.

	ND	HFD	SMV	HFD + R	HFD + C	HFD + RC	HFD + RCC’
Body weight (g)	29.28 ± 5.8 **	51.93 ± 2.19	45.85 ± 5.76	48.48 ± 1.58 *	45.31 ± 6.22 *	44.3 ± 5.7 *	44.68 ± 3.07 **
Food intake (g/week)	16.16 ± 0.8 *	17.94 ± 0.61	16.64 ± 0.42 *	16.14 ± 0.56	16.35 ± 0.38	15.73 ± 0.67	15.46 ± 0.56
Food efficiency ratio	0.021	0.096	0.063	0.076	0.053	0.053	0.061
Total fat (g)	1.06 ± 0.14 **	4.73 ± 0.35	4.26 ± 0.39 *	3.76 ± 0.52 *	3.71 ± 0.3 **	3.64 ± 0.24 **	3.69 ± 0.25 *
Perirenal fat (g)	0.32 ± 0.11 **	1.41 ± 0.2	1.37 ± 0.23	1.13 ± 0.12 *	1.06 ± 0.12 **	0.96 ± 0.16 **	1.04 ± 0.14 *
Abdominal fat (g)	0.21 ± 0.09 **	1.81 ± 0.17	1.51 ± 0.2 *	1.39 ± 0.26 *	1.35 ± 0.1 **	1.39 ± 0.08 **	1.37 ± 0.23 **
Epididymal fat (g)	0.49 ± 0.12 **	1.50 ± 0.18	1.37 ± 0.16	1.24 ± 0.28 *	1.28 ± 0.19 *	1.27 ± 0.11 *	1.23 ± 0.16 *
Liver weight (g)	0.93 ± 0.06	2.41 ± 0.19	1.61 ± 0.34	1.86 ± 0.2	1.70 ± 0.39	1.63 ± 0.31	1.52 ± 0.32

Final body weight (g), food intake (g/week). Food efficiency ratio = body weight gain (g/day)/food intake (g/day), total fat (g), perirenal fat (g), abdominal fat (g), epididymal (g), and liver weight (g), Statistical difference was analyzed using the independent samples *t*-test. Values are represented as mean ± SD (*n* = 7). The results were compared to the high-fat diet (HFD) group using the Student’s *t*-test (** corresponds to *p* < 0.005, * corresponds to *p* < 0.05).

**Table 2:** Effect of *Rubus crataegifolius* extracts on plasma anti-coagulation time.

Treatment	Dose mg/mL	Clotting Time (s)		
		APTT	PT	TT
Control		48 ± 2.2	22 ± 0.07	19.8 ± 2.2
Rubus	0.6	73 ± 3.6	37 ± 2.3	22.2 ± 1.5
	1.25	>1000	>350	>500
	2.5	>1000	>350	>500
	5	>1000	>350	>500
Aspirin	2	73 ± 3.6	37 ± 2.3	22.2 ± 1.5

Data were expressed as mean ± SEM (*n* = 5).

## Data Availability

The data presented in this study are available on request from the corresponding author.

## Sample Availability

Samples of the compounds are available from the authors.
